# Supplementary figures and images for: Parenting beliefs and psychological distress → stimulation and punishment → young children’s behavior: A descriptive study in Colombia
Source: PLoS One. 2025 Nov 6;20(11):e0336106. doi: 10.1371/journal.pone.0336106 (PMC12591459; doi:10.1371/journal.pone.0336106)

**Figure S1.**

*Engagement in different stimulation activities*


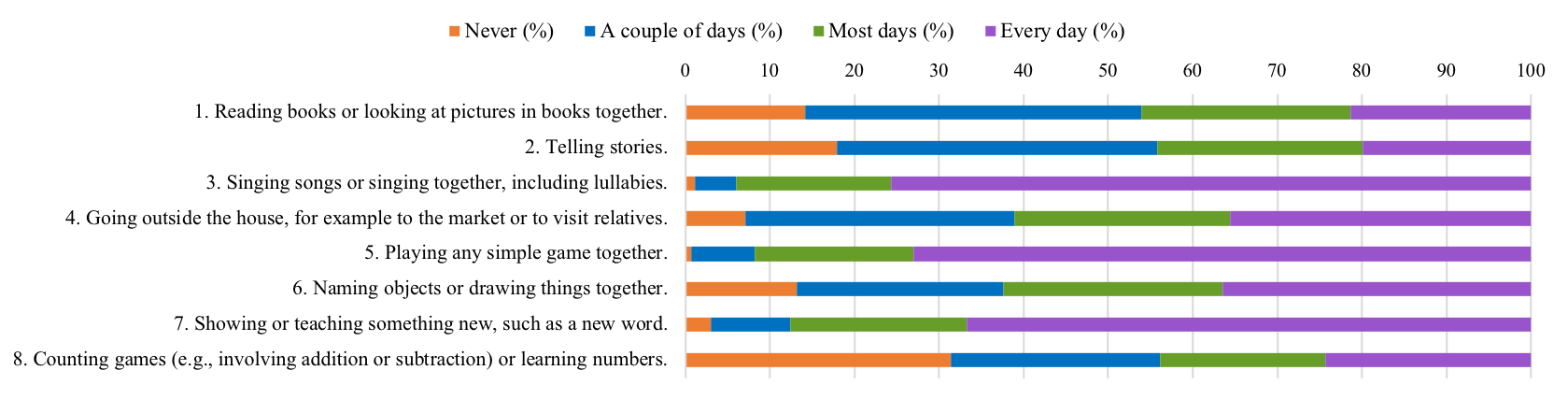

Supplement: S1 Fig — (DOCX) [file pone.0336106.s001.docx]

**Figure S2.**

Use of different punishment methods


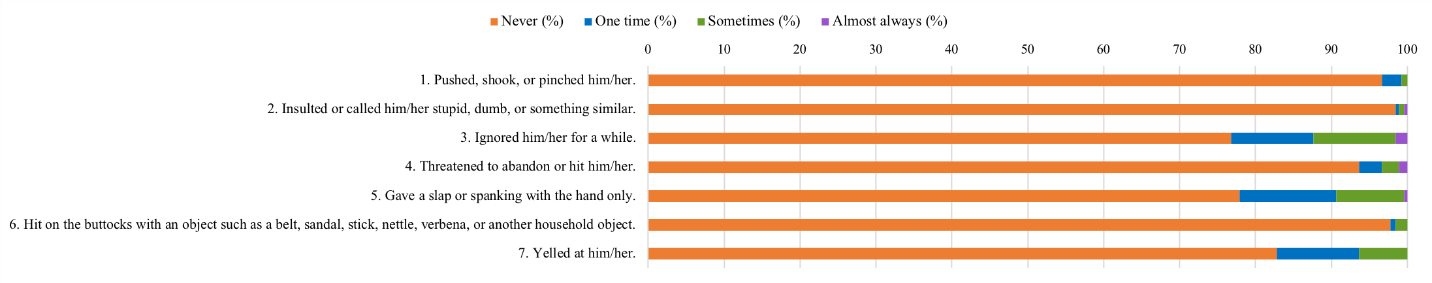

Supplement: S2 Fig — (DOCX) [file pone.0336106.s002.docx]
